# Depressive Symptoms and 24-Hour Ambulatory Blood Pressure in Africans: The SABPA Study

**DOI:** 10.1155/2012/426803

**Published:** 2011-10-19

**Authors:** Mark Hamer, Nancy Frasure-Smith, François Lespérance, Brian H. Harvey, Nico T. Malan, Leoné Malan

**Affiliations:** ^1^Psychobiology Group, Department of Epidemiology and Public Health, University College London, 1-19 Torrington Place, London WC1E 6BT, UK; ^2^Department of Psychiatry and School of Nursing, McGill University CHUM Research Center, QC, Canada; ^3^Montreal Heart Institute Research Center, University of Montreal, Montreal, QC, Canada H3C 3J7; ^4^Unit for Drug Research and Development, Division of Pharmacology, School of Pharmacy, North-West University, Potchefstroom 2520, South Africa; ^5^Hypertension in Africa Research Team (HART), School for Physiology, Nutrition, and Consumer Sciences, North-West University, Potchefstroom 2520, South Africa

## Abstract

Disturbances in circadian rhythm might play a central role in the neurobiology of depression. We examined the association between depressive symptoms and 24-hour ambulatory BP in a sample of 405 (197 black and 208 Caucasian) urbanized African teachers aged 25 to 60 yrs (mean 44.6 ± 9.6 yrs). Depressive symptoms were assessed using the self-administered 9-item Patient Health Questionnaire (PHQ-9). After adjusting for age, sex, and ethnicity, participants with severe depressive symptoms (PHQ-9 ≥ 15) had higher odds of hypertension defined from ambulatory BP and/or use of antihypertensive medication (odds ratio = 2.19, 95% CI, 1.00–4.90) in comparison to participants with no symptoms. Compared to Caucasians with no depressive symptoms, those with severe symptoms had blunted nocturnal systolic BP drop of 4.7 mmHg (95% CI, −0.5 to 10.0, *P* = 0.07). In summary, depressive symptoms were associated with the circadian BP profile in black and Caucasian Africans.

## 1. Introduction

Studies examining the association between common mood disorder, such as depression and risk of hypertension, have produced mixed findings. Several studies have reported positive associations between depression and risk of incident hypertension [1–4] whereas others have observed null findings [5, 6], or attributed the effects to hypertension labeling [7]. There is even some evidence to suggest lower blood pressure (BP) in participants with depressive symptoms [8, 9]. In the Whitehall II study of British civil servants that examined longitudinal trajectories, the risk of hypertension increased with repeated experience of depressive episodes over time and became evident in later adulthood [10]. Thus, the association between depression and hypertension is complex. 

One limitation of this body of work is the reliance on clinic BP readings taken on one occasion. In a recent study of older participants that underwent 24-hour ambulatory BP recordings, those reporting depressive symptoms demonstrated a blunted nocturnal systolic BP fall and were more likely to be nondippers, defined as having nocturnal systolic BP fall of less than 10% [11]. There is increasing recognition that disturbances in circadian rhythm play a central role in the neurobiology of depression, even recognizing its importance as a novel pharmacological target in treating the disorder [12]. Depression is associated with alterations in hormone and catecholamine circadian rhythms [13], which may be the result of poorly regulated neurotransmitter feedback control systems that become erratic when chronically stressed. Melatonin, for example, is an endogenous antihypertensive molecule released by the pineal gland that plays a role in the biological regulation of circadian rhythms and has been associated with depression. In addition, nocturnal melatonin secretion is impaired among nondipper hypertensive patients [14]. 

Further studies are required to examine the association between depressive symptoms and the circadian BP profile, which might help to further clarify the equivocal nature of existing literature. The aim of this study was therefore to examine the association between depressive symptoms and circadian BP profiles using 24-hour ambulatory monitoring. The study was conducted in a sample of black and Caucasian Africans who were recruited as part of the Sympathetic Activity and Ambulatory Blood Pressure in Africans (SABPA) study—presently the only study in sub-Saharan Africa focusing on the contribution of psychosocial risk factors to cardiovascular health. Thus, a further aim was to assess potential ethnic differences in the association between depression and BP.

## 2. Methods

### 2.1. Participants and Procedures

Participants were recruited as part of the SABPA study conducted between February 2008 and May 2009. The study sample comprised urbanized African teachers working for the Dr. Kenneth Kaunda Education district in the North West Province, South Africa. The reason for this selection was to obtain a homogenous sample from a similar socioeconomic class. All eligible participants between the ages of 25 and 60 years were invited to participate. Exclusion criteria were an oral temperature above 37°C, psychotropic substance dependence or abuse, blood donors, and individuals vaccinated in the past 3 months. Participants were fully informed about the objectives and procedures of the study prior to their recruitment. Assistance was available for any participant who requested conveyance of information in their home language. All participants signed an informed consent form and the study was approved by The Ethics Review Board of the North-West University (Potchefstroom Campus). Participants were transported at 1630 hours to the Metabolic Unit Research Facility of the North-West University and familiarized with the protocol and completed a battery of psychosocial questionnaires. After receiving a standardized dinner participants were encouraged to go to bed at around 2200 hours and were woken at 0545 hours the following morning to undergo a battery of clinical assessments. 

### 2.2. Depressive Symptoms

Depressive symptoms were assessed using the self-administered 9-item Patient Health Questionnaire (PHQ-9), which has been extensively validated in various clinical patient groups and different ethnic populations including sub-Saharan Africans [15, 16]. In the present study the PHQ-9 displayed good reliability (Cronbach's alpha = 0.81). The PHQ-9 measures the frequency of depressive symptoms corresponding to the 9 key symptoms of depression used by the *Diagnostic and Statistical Manual of Mental Disorder Fourth Edition* (DSM-IV) criteria to diagnosed major depressive episode. Participants indicated the frequency of experiencing symptoms during the prior two weeks, ranging from “not at all” (scored as zero), “several days” (one point), “more than half the days” (two points), “nearly every day” (three points), thus giving possible scores of 0–27. We used previously described PHQ-9 cut points (15) to derive three categories; no depressive symptoms (score <5), mild-moderate symptoms (score of 5–14), and severe symptoms (score of 15 and above).

### 2.3. Blood Pressure Measures

Semirecumbent clinic BP was measured twice using a mercury Sphygmomanometer with a 5-minute rest between each reading. Participants underwent 24-h ambulatory BP monitoring during the working day prior to the clinic visit. At ~0800 hours, participants were attached with an ambulatory BP monitor (Meditech CE120 CardioTens; Meditech, Budapest, Hungary) on the nondominant arm at their workplace. The apparatus was programmed to measure BP at 30 min intervals during the day (0800–2200 hours) and every hour during nighttime (2200–0600 hours). On average, the cuff successfully inflated 72.6% of the time across all participants. Participants were asked to continue with normal daily activities and record any abnormalities such as headache, nausea, and feeling stressed on their ambulatory diary cards. The data was analyzed using the CardioVisions 1.15 Personal Edition software (Meditech). Hypertension was defined as mean 24-hour BP >125/80 mmHg [17], and nondippers were defined as participants having nocturnal systolic BP fall of less than 10% of their average daytime BP [18].

### 2.4. Covariates

Sodium fluoride, plasma, and serum samples from fasting blood were stored at −80°C for the analysis of biochemical risk markers. Fasting sodium fluoride (glucose) and serum samples for total and high-density lipoprotein (HDL) cholesterol, gamma glutamyl transferase, and high-sensitivity C-reactive protein (CRP) were analyzed using two sequential multiple analyzers (Konelab 20i; Thermo Scientific, Vantaa, Finland; Unicel DXC 800—Beckman and Coulter, Germany). The intra- and intercoefficients of variation for all assays was below 10%. 

### 2.5. Statistical Analyses

Differences in the characteristics of participants in relation to depressive symptoms were analysed using ANOVA tests to examine continuous variables and chi-square tests to examine categorical variables. All CRP values were log transformed to normalize the distribution. General linear models were used to examine differences in BP between depressive symptoms categories. Logistic regression was used to examine the associations of depressive symptoms with hypertension and nondipper status. Hypertension was defined from BP readings and/or use of antihypertensive medication. The models were adjusted for covariates, including age, sex, ethnicity, CRP, body mass index, total/HDL cholesterol, and glucose. These covariates were selected on an *a priori* basis, as depressive behavior is accompanied by perturbations in glucose and lipid metabolism, inflammatory, and neural serotonergic system function, all of which are associated with exacerbated hypertension [19]. We fitted interaction terms in order to assess potential ethnic differences in the association between depression and BP. All analyses were conducted using SPSS version 14.

## 3. Results

The sample comprised 197 black (aged 44.2 ± 8.0 yrs) and 208 Caucasians (aged 45.0 ± 10.8 yrs). Severe depressive symptoms were reported in 11.6% of the sample. Black participants were more likely to report any depressive symptoms compared with Caucasians (age- and sex-adjusted odds ratio = 3.72, 95% CI, 2.35–5.89). Depressive symptoms were associated with several risk factors including higher body mass index and elevated CRP (see Table 1). In addition, black ethnicity was strongly related to other risk factors, including elevated CRP, BMI, and BP.

**Figure 1:**
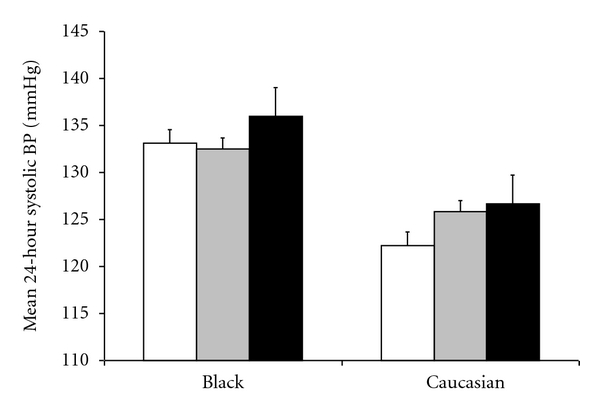
Association between depressive symptoms and mean 24-hour ambulatory systolic blood pressure in black and Caucasian Africans. White, grey, and black bars represent none, mild-moderate, and severe depressive symptoms, respectively. Values for blood pressure are mean ± SEM, adjusted for age, sex, and antihypertensive medication.

Hypertension was prevalent in 62.1% of the sample when using the 24-hour ambulatory BP criteria. There was a strong correlation between clinic and ambulatory systolic BP (*r* = 0.74, *P* < 0.001). Black participants recorded higher 24-hour systolic BP (age-, sex-, medication-adjusted *β* = 7.8, 95% CI, 5.0–10.6 mmHg) compared to Caucasians. There was an association between depressive symptoms and 24-hour ambulatory BP (see Figure 1). For example, compared to participants with no depressive symptoms, those with severe symptoms had marginally increased 24-hour systolic BP of 4.2 mmHg (95% CI, −0.5 to 8.9, *P* = 0.08) after adjustment for age, sex, ethnicity, and antihypertensive medication. Depressive symptoms were associated with a twofold risk of hypertension defined from 24-hour ambulatory BP monitoring (Table 2), although when hypertension was defined from clinic BP readings (≥140/90 mmHg) there was no associations with depression (odds ratio of hypertension for severe depressive symptoms = 1.36, 95% CI, 0.63–2.96). The associations of depressive symptoms and 24-hour hypertension were slightly attenuated after further adjustments for other risk factors including body mass index, CRP, total/HDL cholesterol, and glucose. We found no evidence for any statistically significant ethnic differences in the association between depression and BP. The removal of four participants who reported use of antidepressant medication did not alter any of the results.

Fifty-nine participants (14.5%) recorded experiencing stress on their diary cards during the ambulatory monitoring day, and those participants had elevated 24-hour systolic BP compared with nonstressed (age-, sex-, ethnicity-adjusted *β* = 5.5, 95% CI, 1.7 to 9.3 mmHg, *P* = 0.004). Participants reporting severe depressive symptoms were more likely to report feeling stressed (age-, sex-, ethnicity-adjusted odds ratio = 2.96, 95% CI, 1.16–7.50). When we added stress as a covariate, the difference in 24-hour systolic BP between participants reporting none and severe depressive symptoms was no longer evident (*β* = 3.3, 95% CI, −1.4 to 8.0 mmHg, *P* = 0.16), which suggests stress might partly explain the link between depression and the circadian BP profile.

**Figure 2:**
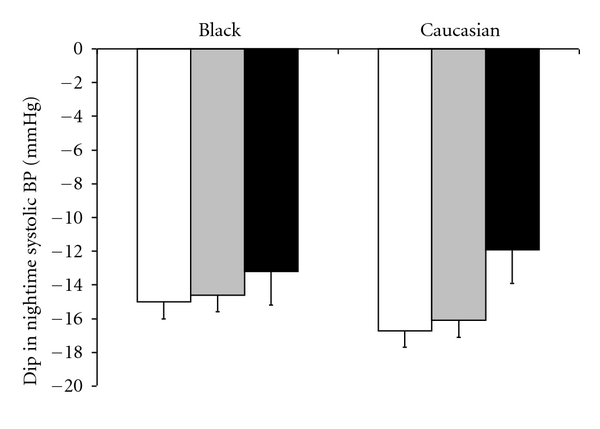
Association between depressive symptoms and change in ambulatory systolic blood pressure between daytime and night in black and Caucasian Africans. White, grey, and black bars represent none, mild-moderate, and severe depressive symptoms, respectively. Values for blood pressure are mean ± SEM, adjusted for age, sex, and antihypertensive medication.

We further examined the association between depressive symptoms and nocturnal BP decline (Table 3 and Figure 2). A nocturnal systolic BP fall of less than 10% (nondipper) was observed in 31.4% of the sample, and black participants were more likely to be nondippers compared with Caucasians (age-, sex-, medication-adjusted odds ratio = 1.62, 95% CI, 1.04–2.51). There was a weak association between depressive symptoms and nocturnal BP decline that was particularly evident in Caucasians. For example, compared to Caucasians with no depressive symptoms, those with severe symptoms had blunted nocturnal systolic BP drop of 4.7 mmHg (95% CI, −0.5 to 10.0, *P* = 0.07) after adjustment for age, sex, and antihypertensive medication (Figure 2). In the whole sample there was evidence of elevated risk of nondipping in participants with severe depressive symptoms although this did not reach statistical significance (Table 3), nor was there any significant interaction by ethnicity. 

## 4. Discussion

This is presently the only study in sub-Saharan Africa to examine the association between depressive symptoms and risk of hypertension. Black Africans are known to have elevated BP compared with Caucasians [20], and this was confirmed in the present study. Black participants from the present study also demonstrated a higher prevalence of depressive symptoms. Despite this, the association between depressive symptoms and BP remained independent of ethnicity and there was no evidence for any significant ethnic differences in the association between depression and BP.

Previous studies in this area have been limited by only taking clinic BP readings, but in the present study we also collected data from 24-hour ambulatory BP monitoring. Our main findings show an association between depressive symptoms and 24-hour ambulatory BP. To our knowledge, only one other study examining depression has employed ambulatory BP monitoring [11]. They found an association between depressive symptoms and blunted nocturnal systolic BP fall. Our findings are partly consistent with those results although we did not find strong evidence for an association between depression and risk of nondipping, which might be explained by the older age of participants from the previous study. Several large epidemiological cohort studies have found evidence for an association between depression and elevated risk of incident hypertension at followup [1–3, 10]. However, in other studies depressive disorder was associated with lower systolic BP [8, 9], although use of tricyclic antidepressants was associated with greater risk of hypertension [5, 8]. These inconsistencies might therefore be partially explained by inadequate assessments of BP, antidepressant medication, different measures of symptoms and diagnostic criteria for depression, or other confounding factors. 

The associations between depressive symptoms and 24-hour BP were attenuated after further adjustments for other risk factors including body mass index, CRP, cholesterol, and glucose. However, given that these factors might act as intermediate mechanisms on the causal pathway from depression to hypertension, our models may have been overadjusted. For example, depressive symptoms were associated with body mass index and CRP, both of which have been implicated in hypertension risk [21, 22]. Indeed, these mechanisms have also been linked with the association between depression and coronary heart disease [23–25].

From our results we might speculate that daily stress partly explains the link between depression and the circadian BP profile. Participants with severe depressive symptoms were more likely to report feeling stressed on the ambulatory BP monitoring day, which corroborates with observations that depressed individuals are more likely to report events as stressful and to complain and endorse somatic symptoms. Heightened BP reactivity to laboratory-induced stressors has been prospectively associated with a greater risk of hypertension [26], thus if elicited repeatedly in a person's life these responses might be clinically relevant. Moreover, there is also overlapping biology between the neurohormonal control of BP and the subsequent downstream effects of psychological stress, one in particular being the gaseous transmitter and neuroregulator, nitric oxide [27]. Indeed, naturalistic studies have shown profound effects of acute stressors on BP. For example, the World Trade Centre terrorist attacks in New York on 11th September 2001 produced a substantial and sustained increase in the BP of participants from the local community [28]. There was also a 20% increase in systolic BP following a moderate intensity earth quake that struck central Italy, which was followed by a long lasting period of enhanced BP variability and blunted nocturnal BP fall [29]. Work stress has also been associated with elevated 24-hour ambulatory BP [30] although the findings from prospective cohort studies in relation to work stress and hypertension risk have been generally inconsistent [31–36].

The strengths of this study include the unique sample of participants from sub-Saharan Africa, a well-validated measure of depressive symptoms, and the use of 24-hour ambulatory BP assessments. A limitation is the cross-sectional design, thus we cannot infer causality, identify potential mediators, nor determine the direction of the observed relationship between depression and hypertension. In addition, we did not assess history of major depression and recurrent symptoms, which might be important given the link between persistent depressive symptoms and cardiovascular risk [37]. In summary, we found an association between depressive symptoms and 24-hour ambulatory BP in black and Caucasian Africans. The equivocal nature of the existing work in this area might be explained by reliance on clinic BP readings and failure to capture the circadian BP profile.

## Figures and Tables

**Table 1:** Characteristics of the study sample (*N* = 405).

Variable	No depressive symptoms		Mild- moderate depressive symptoms		Severe depressive symptoms	
	Black (*n* = 42)	Caucasian (*n* = 101)	Black (*n* = 120)	Caucasian (*n* = 95)	Black (*n* = 35)	Caucasian (*n* = 12)
Age (yrs)	43.8 ± 7.3	46.3 ± 10.8	44.7 ± 8.3	43.8 ± 10.7	43.3 ± 7.8	43.3 ± 11.3
Sex (% men)	66.7	58.4	58.8	45.7	34.3*	33.3*
Body mass index (kg·m^2^)	27.7 ± 6.6	27.2 ± 5.4	30.7 ± 7.2	27.7 ± 6.5	31.2 ± 7.1*	29.8 ± 6.7
Total/HDL cholesterol ratio	4.41 ± 1.85	5.21 ± 1.63	4.34 ± 1.58	4.71 ± 1.58	5.08 ± 3.31	5.41 ± 1.69
C-reactive protein (mg/L)^†^	3.95 (54.3)	1.10 (14.1)	5.05 (53.6)	1.90 (26.3)	5.33 (32.5)	2.90 (25.7)*
Glucose (mmol/L)	5.59 ± 2.23	5.80 ± 0.97	5.59 ± 1.64	5.55 ± 0.62	5.92 ± 3.01	5.53 ± 0.50
Antihypertensive medication usage (%)	21.5	8.9	22.5	8.4	14.3	8.3

*P < 0.05 for trend across depressive symptom groups compared within own ethnicity; values displayed as mean ± SD unless stated (^†^non-normally distributed values displayed as median and range).

**Table 2:** The association between depressive symptoms and hypertension defined from 24 hr ambulatory blood pressure readings. (*N* = 405).

Depressive symptoms	Cases/N	Model 1 OR (95% CI)	Model 2 OR (95% CI)
None	80/143	Reference	Reference
Mild-moderate	142/215	1.91 (1.15–3.18)	1.79 (1.02–3.13)
Severe	32/47	2.19 (1.00–4.90)	1.86 (0.76–4.57)

^‡^defined as mean 24 hr BP ≥ 125/80 mmHg and/or use of antihypertensive medication.

Model 1; adjustment for age, sex, ethnicity.

Model 2; adjustment for age, sex, ethnicity, + C-reactive protein, body mass index, total/HDL cholesterol, glucose.

**Table 3:** The association between depressive symptoms and nondipper status.

Depressive symptoms	Cases/N	Model 1 OR (95% CI)	Model 2 OR (95% CI)
None	45/143	Reference	Reference
Mild-moderate	75/215	1.04 (0.65–1.67)	1.01 (0.62–1.63)
Severe	23/47	1.74 (0.85–3.58)	1.74 (0.83–3.63)

Model 1; adjustment for age, sex, ethnicity, antihypertensive medication.

Model 2; adjustment for age, sex, ethnicity, antihypertensive medication + C-reactive protein, BMI, total/HDL cholesterol, glucose.
